# Supplementary material for: The importance of spawning behavior in understanding the vulnerability of exploited marine fishes in the U.S. Gulf of Mexico
Source: PeerJ. 2021 Jul 27;9:e11814. doi: 10.7717/peerj.11814 (PMC8323598; doi:10.7717/peerj.11814)
Supplement: Supplemental Information 1 [file peerj-09-11814-s001.docx]

**Supplementary Material For:**

**The importance of spawning behavior in understanding the vulnerability of exploited marine fishes in the U.S. Gulf of Mexico**

Christopher R. Biggs^1^, William D. Heyman^2^, Nicholas A. Farmer^3^, Shinichi Kobara^4^, Derek G. Bolser^1^, Jan Robinson^5^, Susan K. Lowerre-Barbieri^6^, and Brad E. Erisman^1,*^

**SELECTION OF SPECIES FOR EVALUATION WITH SPAWNING AGGREGATION AND FISHERIES MANAGEMENT CRITERIA**

Cooperative monitoring program for spawning aggregations

in the Gulf of Mexico:

an assessment of existing information, data gaps and research priorities

Chris Biggs^1^, Brad Erisman^1^, William Heyman^2^, Shinichi Kobara^3^, Nick Farmer^4^, Sue Lowerre-Barbieri^5^, Mandy Karnauskas^6^, Jorge Brenner^7^

^1^ Department of Marine Science, University of Texas at Austin, Port Aransas, TX, 78373, USA, ^2^LGL Ecological Research Associates, Inc., 4103 S. Texas Avenue, Suite 211, Bryan, TX, 77802 USA ^3^Department of Oceanography, Texas A&M University, College Station, TX 77843-3146, USA, ^4^ NOAA Fisheries, Southeast Regional Office, 263 13th Ave S., St. Petersburg, FL 33701, ^5^ Fisheries and Aquatic Science Program, School of Forest Resources and Conservation, University of Florida, 7922 North West 71st Street, Gainesville, FL 32653-307, USA,  ^6^ NOAA Fisheries, Southeast Fishery Science Center, 75 Virginia Beach Dr., Miami, FL 33149, USA, ^7^ The Nature Conservancy, Texas Chapter, 1800 Augusta Dr., Suite 240, Houston, TX 77057, USA

Suggested Citation: Biggs, C., B. Erisman, W. Heyman, S.Kobara, N. Farmer, S. Lowerre-Barbieri, M. Karnauskas, and J. Brenner. (2017). Species selection for evaluation with spawning aggregation and fisheries management criteria. Cooperative monitoring program for spawning aggregations in the Gulf of Mexico. Version 2017.4. Available from GCOOS Web site: http://geo.gcoos.org/restore/selection_criteria

**Selection of Species for Evaluation with Spawning Aggregation and Fisheries Management Criteria**

**Initial List of Species**

A preliminary list of 63 estuarine, coastal, and marine fishes occurring in the Gulf of Mexico (GOM) was compiled for the process to determine which species would be included in the assessment (Table 1). The initial list included all federally managed species, as well as commonly occurring and commonly caught recreational or commercial species that inhabit offshore, coastal, or inshore waters of the GOM. Particular consideration was given to species that are known to form spawning aggregations or species that potentially aggregate to spawn as determined by the expert opinion of the principal investigators and collaborators involved in this project. Highly migratory, schooling, pelagic species (e.g. tunas) were excluded, because as a group, they are well studied and managed as a separate unit. Sharks and other elasmobranchs were also eliminated, because their reproductive patterns do not fit the criteria for spawning aggregations (Domeier 2012 – see Appendix 1). In order to identify the species that would be the most beneficial to include in the assessment, a hierarchical scoring system was created.

**Table 1** List of initial species considered for assessment and analysis.

| **Almaco Jack** | *Seriola rivoliana* | **Mutton Snapper** | *Lutjanus analis* |
| --- | --- | --- | --- |
| **Anchor Tilefish (Gulf Bareye Tilefish)** | *Caulolatilus intermedius* | **Nassau Grouper** | *Epinephelus striatus* |
| **Banded Rudderfish** | *Seriola zonata* | **Permit** | *Trachinotus falcatus* |
| **Bank Sea Bass** | *Centorpristis ocyurus* | **Queen Snapper** | *Etelis oculatus* |
| **Black Drum** | *Pogonias cromis* | **Red Drum** | *Sciaenops ocellatus* |
| **Black Grouper** | *Mycteroperca bonaci* | **Red Grouper** | *Epinephelus morio* |
| **Black Sea Bass** | *Centropristis striata* | **Red Hind** | *Epinephelus guttatus* |
| **Blackfin Snapper** | *Lutjanus buccanella* | **Red Porgy** | *Pagrus pagrus* |
| **Blackline Tilefish** | *Caulolatilus cyanops* | **Red Snapper** | *Lutjanus campechanus* |
| **Bluefish** | *Pomatomus saltatrix* | **Sand Tilefish** | *Malacanthus plumieri* |
| **Blueline Tilefish** | *Caulolatilus microps* | **Scamp** | *Mycteroperca phenax* |
| **Bonefish** | *Abula vulpes* | **Schoolmaster** | *Lutjanus apodus* |
| **Cero** | *Scomberomorus regalis* | **Sheepshead** | *Archosargus probatocephalus* |
| **Chub** | *Kyphosus saltatrix* | **Silk Snapper** | *Lutjanus vivanus* |
| **Cobia** | *Rachycentron canadum* | **Snowy Grouper** | *Hyporthodus niveatus* |
| **Common Snook** | *Centropomus undecimalis* | **Southern Flounder** | *Paralichthys lethostigma* |
| **Cubera Snapper** | *Lutjanus cyanopterus* | **Spanish Mackerel** | *Scomberomorus maculatus* |
| **Dolphinfish** | *Coryphaena hippurus* | **Speckled Hind** | *Epinephelus drummondhayi* |
| **Gag Grouper** | *Mycteroperca microlepis* | **Spotted Seatrout** | *Cynoscion nebulosus* |
| **Goldface Tilefish** | *Caulolatilus chrysops* | **Striped Mullet** | *Mugil cephalus* |
| **Atlantic Goliath Grouper** | *Epinephelus itajara* | **Tarpon** | *Megalops atlanticus* |
| **Gray Snapper** | *Lutjanus griseus* | **Tiger Grouper** | *Mycteroperca tigris* |
| **Gray Triggerfish** | *Balistes capriscus* | **Tilefish** | *Lopholatilus chamaeleonticeps* |
| **Greater Amberjack** | *Seriola dumerili* | **Vermilion Snapper** | *Rhomboplites aurorubens* |
| **Gulf Menhaden** | *Brevoortia patronus* | **Warsaw Grouper** | *Hyporthodus nigritus* |
| **Hogfish** | *Lachnolaimus maximus* | **Wenchman** | *Pristipomoides aquilonaris* |
| **King Mackerel** | *Scomberomorus cavalla* | **Western Comb Grouper** | *Mycteroperca acutirostris* |
| **Lane Snapper** | *Lutjanus synagris* | **Yellowedge Grouper** | *Hyporthodus flavolimbatus* |
| **Lesser Amberjack** | *Seriola fasciata* | **Yellowfin Grouper** | *Mycteroperca venenosa* |
| **Little Tunny** | *Euthynnus alletteratus* | **Yellowmouth Grouper** | *Mycteroperca interstitialis* |
| **Marbled Grouper** | *Dermatolepis inermis* | **Yellowtail Snapper** | *Ocyurus chrysurus* |
| **Misty Grouper** | *Hyporthodus mystacinus* |  |  |

**Scored Criteria**

Species were scored across six criteria based on their aggregation behavior, two aspects of management status, importance to commercial fisheries, importance to recreational fisheries, and their endangered status according to the IUCN Red List (Table 2). Descriptions of each criterion are below.

1. **FSA Score:** The fish spawning aggregation score (FSA score) reflected the degree to which the species aggregates to spawn on a scale of 1-4. Scores of 1 were assigned to species that do not aggregate to spawn, are “simple migratory” spawners (Domeier 2012), or if it is unknown whether they aggregate to spawn. A score of 2 was assigned to species that form resident aggregations, 3 for mixed (resident/transient) aggregations and 4 for strictly transient aggregations. The designations were based on the expert opinion of the principal investigators and collaborators involved in the project.
2. **FMP:** Species that are currently included in the Fishery Management Plan (FMP) of the Gulf of Mexico Fishery Management Council received a score of 1 and those that are not received a score of 0.
3. **FSSI:** The Fish Stock Sustainability Index (FSSI) measures the performance of important commercial and recreational fish stocks, which represent 85% of the total catch. Species that are included in the index received a score of 1 and species that are not included were scored 0.
4. **Commercial Landings:** The average commercial landings (lbs.) in the Gulf of Mexico 2009-2013.
5. **Recreational Landings:** The average recreational landings (number of fish (N)) in the Gulf of Mexico 2009-2013. Data included headboat, charter and private totals from NOAA’s Marine Recreational Information Program (MRIP) and Texas Parks and Wildlife Department (TPWD).

1. **IUCN Red List:** The status of each species on the International Union for Conservation of Nature’s Red List of Threatened Species was accounted for. The status of each species was scored based on the following scale: 1= Least Concern, 2= Near Threatened, 3= Vulnerable, 4= Endangered, 5= Critically Endangered.

**Table 2** Species scores on selection criteria. FSA scores were based on expert opinion of Principal Investigators and collaborators.

| **Common Name** | **FSA score (1-4)** | **FMP**  **(y=1,n=0)** | **FSSI**  **(y=1,n=0)** | **Avg. comm. landings**  **(2009-2013) (lbs)** | **Avg. rec. landings (2009-2013) (N)** | **IUCN** |
| --- | --- | --- | --- | --- | --- | --- |
| Almaco Jack | 3 | 1 | 0 | 36277 | 15341 | 1 |
| Anchor Tilefish  (Gulf Bareye Tilefish) | 1 | 0 | 0 | 0 | 0 | 1 |
| Banded Rudderfish | 1 | 1 | 0 | 17549 | 70765 | 1 |
| Bank Sea Bass | 1 | 0 | 0 | 0 | 12067 | 1 |
| Black Drum | 3 | 0 | 0 | 0 | 0 | 1 |
| Black Grouper | 4 | 1 | 1 | 46855 | 5530 | 2 |
| Black Sea Bass | 1 | 0 | 0 | 0 | 1474271 | 1 |
| Blackfin Snapper | 4 | 1 | 0 | 4698 | 142 | 0 |
| Blackline Tilefish | 1 | 0 | 0 | 0 | 0 | 1 |
| Bluefish | 1 | 1 | 0 | 0 | 0 | 3 |
| Blueline Tilefish | 1 | 1 | 0 | 67901 | 238 | 0 |
| Bonefish | 3 | 1 | 0 | 0 | 0 | 2 |
| Cero | 1 | 1 | 0 | 0 | 0 | 1 |
| Chub | 2 | 0 | 0 | 0 | 0 | 1 |
| Cobia | 1 | 1 | 0 | 69204 | 95062 | 1 |
| Common Snook | 3 | 1 | 0 | 0 | 0 | 1 |
| Cubera Snapper | 4 | 1 | 0 | 1307 | 929 | 3 |
| Dolphinfish | 1 | 1 | 0 | 134154 | 693780 | 1 |
| Gag Grouper | 3 | 1 | 1 | 620534 | 1835929 | 1 |
| Goldface Tilefish | 1 | 1 | 0 | 9056 | 7 | 1 |
| Atlantic Goliath Grouper | 4 | 1 | 0 | 0 | 3 | 5 |
| Gray Snapper | 3 | 1 | 0 | 155194 | 4299334 | 0 |
| Gray Triggerfish | 2 | 1 | 1 | 74997 | 364994 | 3 |
| Greater Amberjack | 3 | 1 | 1 | 481954 | 272351 | 1 |
| Gulf Menhaden | 1 | 1 | 0 | 0 | 0 | 1 |
| Hogfish | 2 | 1 | 1 | 36203 | 133271 | 3 |
| King Mackerel | 3 | 1 | 0 | 3604244 | 403641 | 1 |
| Lane Snapper | 3 | 1 | 0 | 23923 | 394839 | 0 |
| Lesser Amberjack | 1 | 1 | 0 | 21190 | 952 | 1 |
| Little Tunny | 3 | 1 | 0 | 0 | 0 | 1 |
| Marbled Grouper | 4 | 0 | 0 | 0 | 0 | 2 |
| Misty Grouper | 1 | 0 | 0 | 163 | 1 | 1 |
| Mutton Snapper | 4 | 1 | 0 | 77736 | 3956 | 3 |
| Nassau Grouper | 4 | 0 | 1 | 0 | 0 | 5 |
| Permit | 3 | 1 | 0 | 0 | 0 | 1 |
| Queen Snapper | 4 | 1 | 0 | 12427 | 66 | 0 |
| Red Drum | 3 | 1 | 1 | 0 | 0 | 1 |
| Red Grouper | 3 | 1 | 1 | 4992180 | 2657260 | 2 |
| Red Hind | 4 | 0 | 0 | 1553 | 3663 | 1 |
| Red Porgy | 1 | 0 | 0 | 0 | 451431 | 1 |
| Red Snapper | 2 | 1 | 1 | 3773741 | 2812127 | 3 |
| Sand Tilefish | 1 | 0 | 0 | 0 | 896 | 1 |
| Scamp | 3 | 1 | 0 | 246538 | 70454 | 1 |
| Schoolmaster | 4 | 0 | 0 | 88 | 3343 | 0 |
| Sheepshead | 3 | 0 | 0 | 0 | 0 | 1 |
| Silk Snapper | 3 | 1 | 0 | 38597 | 26382 | 0 |
| Snowy Grouper | 3 | 1 | 1 | 153962 | 9102 | 3 |
| Southern Flounder | 3 | 0 | 0 | 0 | 0 | 2 |
| Spanish Mackerel | 3 | 1 | 0 | 1506135 | 4298114 | 1 |
| Speckled Hind | 1 | 1 | 0 | 41720 | 1311 | 5 |
| Spotted Seatrout | 2 | 1 | 0 | 0 | 0 | 1 |
| Striped Mullet | 3 | 0 | 0 | 0 | 0 | 1 |
| Tarpon | 1 | 1 | 0 | 0 | 0 | 3 |
| Tiger Grouper | 4 | 0 | 0 | 0 | 0 | 1 |
| Tilefish | 3 | 1 | 0 | 376649 | 876579 | 4 |
| Vermilion Snapper | 2 | 1 | 1 | 2581867 | 10084 | 0 |
| Warsaw Grouper | 3 | 1 | 0 | 97402 | 943 | 5 |
| Wenchman | 1 | 1 | 0 | 30465 | 0 | 1 |
| Western Comb Grouper | 1 | 0 | 0 | 0 | 0 | 1 |
| Yellowedge Grouper | 3 | 1 | 1 | 742028 | 656 | 3 |
| Yellowfin Grouper | 4 | 1 | 0 | 1511 | 801 | 2 |
| Yellowmouth Grouper | 4 | 1 | 0 | 421 | 194 | 3 |
| Yellowtail Snapper | 2 | 1 | 0 | 718060 | 10883 | 0 |

**The Selection Process**

The first priority was to keep all species that are included in the FSSI, because these species have previously been identified by NOAA Fisheries as especially important to the commercial and recreational fisheries of the GOM. Twelve species were selected in this step and removed from the pool (Table 3).

**Table 3** Fish Stock Sustainability Index (FSSI) species selected for analysis and removed from the potential selection pool.

| **Common Name** | **FSA score (1-4)** | **FMP (y=1,n=0)** | **FSSI (y=1,n=0)** | **Avg. comm. landings**  **(2009-2013) (lbs)** | **Avg. rec. landings**  **(2009-2013)**  **(N)** | **IUCN** |
| --- | --- | --- | --- | --- | --- | --- |
| Black Grouper | 4 | 1 | 1 | 46855 | 5530 | 2 |
| Gag Grouper | 3 | 1 | 1 | 620534 | 1835929 | 1 |
| Gray Triggerfish | 2 | 1 | 1 | 74997 | 364994 | 3 |
| Greater Amberjack | 3 | 1 | 1 | 481954 | 272351 | 1 |
| Hogfish | 2 | 1 | 1 | 36203 | 133271 | 3 |
| Nassau Grouper | 4 | 0 | 1 | 0 | 0 | 5 |
| Red Drum | 3 | 1 | 1 | 0 | 0 | 1 |
| Red Grouper | 3 | 1 | 1 | 4992180 | 2657260 | 2 |
| Red Snapper | 2 | 1 | 1 | 3773741 | 2812127 | 3 |
| Snowy Grouper | 3 | 1 | 1 | 153962 | 9102 | 3 |
| Vermilion Snapper | 2 | 1 | 1 | 2581867 | 10084 | 0 |
| Yellowedge Grouper | 3 | 1 | 1 | 742028 | 656 | 3 |

*Fishery Index*

To evaluate the commercial and recreational landings data, a commercial rank (Comm. rank) and recreational rank (Rec. rank) value was created based on the respective average landings. Commercial landings data was available for 37 species, so values were assigned from 1.01 for the least caught fish, to 1.37 for the most caught fish. Recreational landings data was available for 41 species, so rank values ranged from 1.01 for the least caught species to 1.41 for the most caught. Species with no landings received a score of 0.

To capture the importance of each remaining species to fisheries, scores from the categories FMP, IUCN, Comm. rank, and Rec. rank were summed to provide a fishery index value. Species within the top half of scores were kept (scores 8.35 – 4.24), and species from the bottom half (< 4.24) of the list were removed, reducing the potential pool to 16 species (Table 4). The fishery index was then added to the FSA value to give an additional score. Species in the top half of values were selected (scores 11.35-7.38) and the remaining species were removed from the selection pool.

**Table 4** Species scored by the fishery index (sum of FMP, FSSI, IUCN, Comm. rank & Rec. rank) and the fishery index plus FSA score. Orange indicates species that were selected for inclusion in analysis.

| **Common Name** | **FSA score (1-4)** | **FMP**  **(y=1,n=0)** | **FSSI**  **(y=1,n=0)** | **Avg. comm. landings**  **(2009-2013) (lbs)** | **Avg. rec. landings**  **(2009-2013) (N)** | **IUCN** | **Comm. rank** | **Rec. rank** | **Fishery Index (Sum of FMP, FSSI, IUCN, Comm. rank, Rec. rank** | **FSA + Fishery Index** |
| --- | --- | --- | --- | --- | --- | --- | --- | --- | --- | --- |
| Warsaw Grouper | 3 | 1 | 0 | 97402 | 943 | 5 | 1.23 | 1.12 | 8.35 | 11.35 |
| Atlantic Goliath Grouper | 4 | 1 | 0 | 0 | 3 | 5 | 0 | 1.02 | 7.02 | 11.02 |
| Tilefish | 3 | 1 | 0 | 376649 | 876579 | 4 | 1.28 | 1.35 | 7.63 | 10.63 |
| Mutton Snapper | 4 | 1 | 0 | 77736 | 3956 | 3 | 1.22 | 1.17 | 6.39 | 10.39 |
| Cubera Snapper | 4 | 1 | 0 | 1307 | 929 | 3 | 1.04 | 1.11 | 6.15 | 10.15 |
| Yellowmouth Grouper | 4 | 1 | 0 | 421 | 194 | 3 | 1.03 | 1.06 | 6.09 | 10.09 |
| Speckled Hind | 1 | 1 | 0 | 41720 | 1311 | 5 | 1.17 | 1.14 | 8.31 | 9.31 |
| Yellowfin Grouper | 4 | 1 | 0 | 1511 | 801 | 2 | 1.05 | 1.09 | 5.14 | 9.14 |
| Spanish Mackerel | 3 | 1 | 0 | 1506135 | 4298114 | 1 | 1.33 | 1.4 | 4.73 | 7.73 |
| King Mackerel | 3 | 1 | 0 | 3604244 | 403641 | 1 | 1.35 | 1.32 | 4.67 | 7.67 |
| Scamp | 3 | 1 | 0 | 246538 | 70454 | 1 | 1.27 | 1.25 | 4.52 | 7.52 |
| Almaco Jack | 3 | 1 | 0 | 36277 | 15341 | 1 | 1.15 | 1.23 | 4.38 | 7.38 |
| Dolphinfish | 1 | 1 | 0 | 134154 | 693780 | 1 | 1.24 | 1.34 | 4.58 | 5.58 |
| Cobia | 1 | 1 | 0 | 69204 | 95062 | 1 | 1.2 | 1.27 | 4.47 | 5.47 |
| Banded Rudderfish | 1 | 1 | 0 | 17549 | 70765 | 1 | 1.1 | 1.26 | 4.36 | 5.36 |
| Lesser Amberjack | 1 | 1 | 0 | 21190 | 952 | 1 | 1.11 | 1.13 | 4.24 | 5.24 |

This step-wise process produced a list of 24 species to be included in the final assessment. Since our selection process was biased towards federally managed species, the Principal Investigators and collaborators then selected (via a consensus vote) four additional species post hoc (spotted seatrout, sheepshead, southern flounder & black drum) known to be common and important to coastal (state) commercial or recreational fisheries in the Gulf of Mexico. This resulted in a final list of 28 species to be evaluated for the project (Table 5).

**Table 5** Final list of species selected for evaluation with spawning aggregation and fishing vulnerability criteria. Yellow= FSSI selected species, Orange= Fishery index and FSA selected species, Green= species added post hoc

| **Common Name** | **FSA score (1-4)** | **FMP**  **(y=1,n=0)** | **FSSI**  **(y=1,n=0)** | **Avg. comm. landings**  **(2009-2013) (lbs)** | **Avg. rec. landings**  **(2009-2013)**  **(N)** | **IUCN** | **Comm. rank** | **Rec. rank** | **Fishery Index (Sum of FMP, FSSI, IUCN, Comm. rank, Rec. rank)** | **FSA + Fishery Index** |
| --- | --- | --- | --- | --- | --- | --- | --- | --- | --- | --- |
| Black Grouper | 4 | 1 | 1 | 46855 | 5530 | 2 | 1.18 | 1.18 | 6.36 | 10.36 |
| Gag Grouper | 3 | 1 | 1 | 620534 | 1835929 | 1 | 1.3 | 1.37 | 5.67 | 8.67 |
| Gray Triggerfish | 2 | 1 | 1 | 74997 | 364994 | 3 | 1.21 | 1.3 | 7.51 | 9.51 |
| Greater Amberjack | 3 | 1 | 1 | 481954 | 272351 | 1 | 1.29 | 1.29 | 5.58 | 8.58 |
| Hogfish | 2 | 1 | 1 | 36203 | 133271 | 3 | 1.14 | 1.28 | 7.42 | 9.42 |
| Nassau Grouper | 4 | 0 | 1 | 0 | 0 | 4 | 0 | 0 | 6 | 10 |
| Red Drum | 3 | 1 | 1 | 0 | 0 | 1 | 0 | 0 | 3 | 6 |
| Red Grouper | 3 | 1 | 1 | 4992180 | 2657260 | 2 | 1.37 | 1.38 | 6.75 | 9.75 |
| Red Snapper | 2 | 1 | 1 | 3773741 | 2812127 | 3 | 1.36 | 1.39 | 7.75 | 9.75 |
| Snowy Grouper | 3 | 1 | 1 | 153962 | 9102 | 3 | 1.25 | 1.19 | 7.44 | 10.44 |
| Vermilion Snapper | 2 | 1 | 1 | 2581867 | 10084 | 0 | 1.34 | 1.2 | 4.54 | 6.54 |
| Yellowedge Grouper | 3 | 1 | 1 | 742028 | 656 | 3 | 1.32 | 1.08 | 7.4 | 10.4 |
| Warsaw Grouper | 3 | 1 | 0 | 97402 | 943 | 5 | 1.23 | 1.12 | 8.35 | 11.35 |
| Atlantic Goliath Grouper | 4 | 1 | 0 | 0 | 3 | 5 | 0 | 1.02 | 7.02 | 11.02 |
| Tilefish | 3 | 1 | 0 | 376649 | 876579 | 4 | 1.28 | 1.35 | 7.63 | 10.63 |
| Mutton Snapper | 4 | 1 | 0 | 77736 | 3956 | 3 | 1.22 | 1.17 | 6.39 | 10.39 |
| Cubera Snapper | 4 | 1 | 0 | 1307 | 929 | 3 | 1.04 | 1.11 | 6.15 | 10.15 |
| Yellowmouth Grouper | 4 | 1 | 0 | 421 | 194 | 3 | 1.03 | 1.06 | 6.09 | 10.09 |
| Speckled Hind | 1 | 1 | 0 | 41720 | 1311 | 5 | 1.17 | 1.14 | 8.31 | 9.31 |
| Yellowfin Grouper | 4 | 1 | 0 | 1511 | 801 | 2 | 1.05 | 1.09 | 5.14 | 9.14 |
| Spanish Mackerel | 3 | 1 | 0 | 1506135 | 4298114 | 1 | 1.33 | 1.4 | 4.73 | 7.73 |
| King Mackerel | 3 | 1 | 0 | 3604244 | 403641 | 1 | 1.35 | 1.32 | 4.67 | 7.67 |
| Scamp | 3 | 1 | 0 | 246538 | 70454 | 1 | 1.27 | 1.25 | 4.52 | 7.52 |
| Almaco Jack | 3 | 1 | 0 | 36277 | 15341 | 1 | 1.15 | 1.23 | 4.38 | 7.38 |
| Southern Flounder | 3 | 0 | 0 | 0 | 0 | 2 | 0 | 0 | 2 | 5 |
| Spotted Seatrout | 2 | 1 | 0 | 0 | 0 | 1 | 0 | 0 | 2 | 4 |
| Black Drum | 3 | 0 | 0 | 0 | 0 | 1 | 0 | 0 | 1 | 4 |
| Sheepshead | 3 | 0 | 0 | 0 | 0 | 1 | 0 | 0 | 1 | 4 |

**Appendix I: Spawning Aggregations – Definitions, Scales, Types, and other Factors to Consider**

**Basic Definition from Domeier (2012):**

A spawning aggregation is a repeated concentration of conspecific animals, gathered for the purpose of reproduction, which is predictable in time and space and results in a mass point source of offspring. The density/number of individuals participating in a spawning aggregation is at least **four times** that found outside the aggregation.

- Requires migration and increase in density/abundance
- Meant to exclude simple migrations of groups (ie. Simple Migratory Spawning – migration of pairs or groups of fishes from a non-spawning to a spawning area; Domeier & Colin 1997).
- Meant to exclude a pair spawn plus a streaking (parasitic) male
- Meant to distinguish a phenomenon where an entire subpopulation of individuals halt their normal routine, migrate, gather and spawn.
- The definition and criteria was intended to capture the conservation/fisheries component related to increased catchability leading to a target for fishing (due to large concentrations, predictable in time and space).
- Excludes sites for copulation (e.g. elasmobranchs) since they no offspring are released, but single sex gatherings for the purpose of releasing offspring (e.g. decapods) are considered spawning aggregations.

**Types of Spawning Aggregations (from Nemeth 2009; Domeier 2012):**

1. Resident spawning aggregations draw individuals to a site within or nearby their adult home range. They usually (1) occur at a specific time of day over numerous days, (2) last only a few hours or less, (3) occur daily over an often lengthy reproductive period of the year; and (4) can occur year round. A single day of spawning for an individual participating in a resident spawning aggregation represents a small fraction of that individual’s annual reproductive effort.
2. Transient spawning aggregations draw individuals to a site well outside their typical adult home range. Transient spawning aggregations often (1) occur during a very specific portion of one or two months of the year; (2) persist for a period of days or at most a few weeks and (3) do not occur year-round. A single transient spawning aggregation may represent the total reproductive effort for participating individuals.

- Functional differences to justify separation of resident and transient beyond just scale: separation falls along phylogenetic lines and trophic levels (predators are transient, herbivores mainly resident)
- Acknowledgement of the existence of exceptions and the fact that many show intermediate patterns (partial migrations – e.g. leopard coraltrout).
- Schooling fishes are known to join conspecific schools at predictable sites and times to form both transient (jacks, mackerels, tunas, croakers) and resident (wrasses, parrotfishes) aggregations. The movement of a single school of animals to a specific site to spawn falls under the definition of Simple Migratory Spawning.

**Egg Types and Spawning Aggregations**

- Most release pelagic eggs
- Some are demersal spawners (e.g. Balistids, pomacentrids, siganids)
- Can include those with or without parental care
- Copulating/live bearers/elasmobranchs – see definitions

**Spawning Aggregations in non-reef habitats**

Pelagic fishes and migratory coastal species at higher latitudes (e.g. temperate) appear to be more likely to exhibit less spatial precision than demersal species. Croakers, herrings/sardines, mullets and flyingfishes are examples of fishes that form predictable spawning aggregations on a larger spatial scale.

**Appendix Table A1** Scores for all species in the potential candidate pool.

| **Common Name** | **FSA score (1-4)** | **FMP (y=1, n=0)** | **FSSI (y=1, n=0)** | **Avg. comm. landings**  **(2009-2013) (lbs)** | **Avg. rec. landings (2009-2013) (N)** | **IUCN** | **Comm. rank** | **Rec. rank** | **Fishery Index (Sum of FMP, FSSI, IUCN, Comm. rank, Rec. rank)** | **FSA + Fishery Index** |
| --- | --- | --- | --- | --- | --- | --- | --- | --- | --- | --- |
| **Black Grouper** | **4** | **1** | **1** | **46855** | **5530** | **2** | **1.18** | **1.18** | **6.36** | **10.36** |
| **Gag Grouper** | **3** | **1** | **1** | **620534** | **1835929** | **1** | **1.3** | **1.37** | **5.67** | **8.67** |
| **Gray Triggerfish** | **2** | **1** | **1** | **74997** | **364994** | **3** | **1.21** | **1.3** | **7.51** | **9.51** |
| **Greater Amberjack** | **3** | **1** | **1** | **481954** | **272351** | **1** | **1.29** | **1.29** | **5.58** | **8.58** |
| **Hogfish** | **2** | **1** | **1** | **36203** | **133271** | **3** | **1.14** | **1.28** | **7.42** | **9.42** |
| **Nassau Grouper** | **4** | **0** | **1** | **0** | **0** | **4** | **0** | **0** | **6** | **10** |
| **Red Drum** | **3** | **1** | **1** | **0** | **0** | **1** | **0** | **0** | **3** | **6** |
| **Red Grouper** | **3** | **1** | **1** | **4992180** | **2657260** | **2** | **1.37** | **1.38** | **6.75** | **9.75** |
| **Red Snapper** | **2** | **1** | **1** | **3773741** | **2812127** | **3** | **1.36** | **1.39** | **7.75** | **9.75** |
| **Snowy Grouper** | **3** | **1** | **1** | **153962** | **9102** | **3** | **1.25** | **1.19** | **7.44** | **10.44** |
| **Vermilion Snapper** | **2** | **1** | **1** | **2581867** | **10084** | **0** | **1.34** | **1.2** | **4.54** | **6.54** |
| **Yellowedge Grouper** | **3** | **1** | **1** | **742028** | **656** | **3** | **1.32** | **1.08** | **7.4** | **10.4** |
| **Warsaw Grouper** | **3** | **1** | **0** | **97402** | **943** | **5** | **1.23** | **1.12** | **8.35** | **11.35** |
| **Atlantic Goliath Grouper** | **4** | **1** | **0** | **0** | **3** | **5** | **0** | **1.02** | **7.02** | **11.02** |
| **Tilefish** | **3** | **1** | **0** | **376649** | **876579** | **4** | **1.28** | **1.35** | **7.63** | **10.63** |
| **Mutton Snapper** | **4** | **1** | **0** | **77736** | **3956** | **3** | **1.22** | **1.17** | **6.39** | **10.39** |
| **Cubera Snapper** | **4** | **1** | **0** | **1307** | **929** | **3** | **1.04** | **1.11** | **6.15** | **10.15** |
| **Yellowmouth Grouper** | **4** | **1** | **0** | **421** | **194** | **3** | **1.03** | **1.06** | **6.09** | **10.09** |
| **Speckled Hind** | **1** | **1** | **0** | **41720** | **1311** | **5** | **1.17** | **1.14** | **8.31** | **9.31** |
| **Yellowfin Grouper** | **4** | **1** | **0** | **1511** | **801** | **2** | **1.05** | **1.09** | **5.14** | **9.14** |
| **Spanish Mackerel** | **3** | **1** | **0** | **1506135** | **4298114** | **1** | **1.33** | **1.4** | **4.73** | **7.73** |
| **King Mackerel** | **3** | **1** | **0** | **3604244** | **403641** | **1** | **1.35** | **1.32** | **4.67** | **7.67** |
| **Scamp** | **3** | **1** | **0** | **246538** | **70454** | **1** | **1.27** | **1.25** | **4.52** | **7.52** |
| **Almaco Jack** | **3** | **1** | **0** | **36277** | **15341** | **1** | **1.15** | **1.23** | **4.38** | **7.38** |
| **Southern Flounder** | **3** | **0** | **0** | **0** | **0** | **2** | **0** | **0** | **2** | **5** |
| **Spotted Seatrout** | **2** | **1** | **0** | **0** | **0** | **1** | **0** | **0** | **2** | **4** |
| **Black Drum** | **3** | **0** | **0** | **0** | **0** | **1** | **0** | **0** | **1** | **4** |
| **Sheepshead** | **3** | **0** | **0** | **0** | **0** | **1** | **0** | **0** | **1** | **4** |
| **Red Hind** | **4** | **0** | **0** | **1553** | **3663** | **1** | **1.06** | **1.16** | **3.22** | **7.22** |
| **Queen Snapper** | **4** | **1** | **0** | **12427** | **66** | **0** | **1.09** | **1.04** | **3.13** | **7.13** |
| **Blackfin Snapper** | **4** | **1** | **0** | **4698** | **142** | **0** | **1.07** | **1.05** | **3.12** | **7.12** |
| **Gray Snapper** | **3** | **1** | **0** | **155194** | **4299334** | **0** | **1.26** | **1.41** | **3.67** | **6.67** |
| **Lane Snapper** | **3** | **1** | **0** | **23923** | **394839** | **0** | **1.12** | **1.31** | **3.43** | **6.43** |
| **Silk Snapper** | **3** | **1** | **0** | **38597** | **26382** | **0** | **1.16** | **1.24** | **3.4** | **6.4** |
| **Schoolmaster** | **4** | **0** | **0** | **88** | **3343** | **0** | **1.01** | **1.15** | **2.16** | **6.16** |
| **Bonefish** | **3** | **1** | **0** | **0** | **0** | **2** | **0** | **0** | **3** | **6** |
| **Marbled Grouper** | **4** | **0** | **0** | **0** | **0** | **2** | **0** | **0** | **2** | **6** |
| **Dolphinfish** | **1** | **1** | **0** | **134154** | **693780** | **1** | **1.24** | **1.34** | **4.58** | **5.58** |
| **Yellowtail Snapper** | **2** | **1** | **0** | **718060** | **10883** | **0** | **1.31** | **1.21** | **3.52** | **5.52** |
| **Cobia** | **1** | **1** | **0** | **69204** | **95062** | **1** | **1.2** | **1.27** | **4.47** | **5.47** |
| **Banded Rudderfish** | **1** | **1** | **0** | **17549** | **70765** | **1** | **1.1** | **1.26** | **4.36** | **5.36** |
| **Lesser Amberjack** | **1** | **1** | **0** | **21190** | **952** | **1** | **1.11** | **1.13** | **4.24** | **5.24** |
| **Goldface Tilefish** | **1** | **1** | **0** | **9056** | **7** | **1** | **1.08** | **1.03** | **4.11** | **5.11** |
| **Bluefish** | **1** | **1** | **0** | **0** | **0** | **3** | **0** | **0** | **4** | **5** |
| **Tarpon** | **1** | **1** | **0** | **0** | **0** | **3** | **0** | **0** | **4** | **5** |
| **Common Snook** | **3** | **1** | **0** | **0** | **0** | **1** | **0** | **0** | **2** | **5** |
| **Little Tunny** | **3** | **1** | **0** | **0** | **0** | **1** | **0** | **0** | **2** | **5** |
| **Permit** | **3** | **1** | **0** | **0** | **0** | **1** | **0** | **0** | **2** | **5** |
| **Tiger Grouper** | **4** | **0** | **0** | **0** | **0** | **1** | **0** | **0** | **1** | **5** |
| **Blueline Tilefish** | **1** | **1** | **0** | **67901** | **238** | **0** | **1.19** | **1.07** | **3.26** | **4.26** |
| **Wenchman** | **1** | **1** | **0** | **30465** | **0** | **1** | **1.13** | **0** | **3.13** | **4.13** |
| **Misty Grouper** | **1** | **0** | **0** | **163** | **1** | **1** | **1.02** | **1.01** | **3.03** | **4.03** |
| **Striped Mullet** | **3** | **0** | **0** | **0** | **0** | **1** | **0** | **0** | **1** | **4** |
| **Black Sea Bass** | **1** | **0** | **0** | **0** | **1474271** | **1** | **0** | **1.36** | **2.36** | **3.36** |
| **Red Porgy** | **1** | **0** | **0** | **0** | **451431** | **1** | **0** | **1.33** | **2.33** | **3.33** |
| **Bank Sea Bass** | **1** | **0** | **0** | **0** | **12067** | **1** | **0** | **1.22** | **2.22** | **3.22** |
| **Sand Tilefish** | **1** | **0** | **0** | **0** | **896** | **1** | **0** | **1.1** | **2.1** | **3.1** |
| **Cero** | **1** | **1** | **0** | **0** | **0** | **1** | **0** | **0** | **2** | **3** |
| **Gulf Menhaden** | **1** | **1** | **0** | **0** | **0** | **1** | **0** | **0** | **2** | **3** |
| **Chub** | **2** | **0** | **0** | **0** | **0** | **1** | **0** | **0** | **1** | **3** |
| **Anchor Tilefish (Gulf Bareye Tilefish)** | **1** | **0** | **0** | **0** | **0** | **1** | **0** | **0** | **1** | **2** |
| **Blackline Tilefish** | **1** | **0** | **0** | **0** | **0** | **1** | **0** | **0** | **1** | **2** |
| **Western Comb Grouper** | **1** | **0** | **0** | **0** | **0** | **1** | **0** | **0** | **1** | **2** |

**Literature Cited**

Domeier, M. L. (2012). Revisiting Spawning Aggregations: Definitions and Challenges. In Y. Sadovy De Mitcheson & P. L. Colin (Eds.), *Reef fish spawning aggregations: biology* (p. 20). Springer Netherlands. <http://doi.org/10.1007/978-94-007-1980-4_1>

Domeier, M. L., & Colin, P. L. (1997). Tropical Reef Fish Spawning Aggregrations: Defined and Reviewed. *Bulletin of Marine Science*, *60*(3), 698–726. Journal Article. Retrieved from http://www.ingentaconnect.com/content/umrsmas/bullmar/1997/00000060/00000003/art00006

Nemeth, R.S. (2009) Dynamics of reef fish and decapod crustacean spawning aggregations: underlying mechanisms, habitat linkages and trophic interactions. Ch 4. In: Nagelkerken I (ed) Ecological Connectivity among Tropical Coastal Ecosystems. Springer, Dordrecht, p73-134
