# Supplementary material for: The importance of spawning behavior in understanding the vulnerability of exploited marine fishes in the U.S. Gulf of Mexico
Source: PeerJ. 2021 Jul 27;9:e11814. doi: 10.7717/peerj.11814 (PMC8323598; doi:10.7717/peerj.11814)
Supplement: Supplemental Information 2 [file peerj-09-11814-s002.docx]

**Supplementary Material For:**

**The importance of spawning behavior in understanding the vulnerability of exploited marine fishes in the U.S. Gulf of Mexico**

Christopher R. Biggs^1^, William D. Heyman^2^, Nicholas A. Farmer^3^, Shinichi Kobara^4^, Derek G. Bolser^1^, Jan Robinson^5^, Susan K. Lowerre-Barbieri^6^, and Brad E. Erisman^1,*^

**Table S2.1** Spearman’s correlation coefficients (upper diagonal) and p-value (lower diagonal) of spawning behavior and life history parameters. Significant correlation values are in bold.

|  |  | **Correlation Coefficient** | | | | | | | | | | |
| --- | --- | --- | --- | --- | --- | --- | --- | --- | --- | --- | --- | --- |
|  |  | **Agg** | **Duration** | **Rel. Ab.** | ***A_max_*** | ***W_max_*** | ***L_max_*** | ***K*** | ***L_inf_*** | ***A_m_*** | ***L_m_*** | ***M*** |
| **p-value** | **Agg** | NA | -0.351 | **0.819** | -0.110 | 0.174 | 0.037 | -0.028 | 0.189 | 0.202 | 0.358 | -0.089 |
|  | **Duration** | 0.06 | NA | **-0.538** | 0.197 | -0.048 | 0.006 | **-0.526** | 0.195 | 0.191 | 0.087 | 0.012 |
|  | **Rel. Ab.** | 0.00 | 0.01 | NA | -0.192 | 0.077 | -0.050 | 0.203 | -0.015 | 0.062 | 0.220 | 0.005 |
|  | ***A_max_*** | 0.58 | 0.31 | 0.33 | NA | **0.431** | 0.353 | -0.362 | **0.377** | **0.628** | 0.314 | **-0.839** |
|  | ***W_max_*** | 0.38 | 0.81 | 0.70 | 0.02 | NA | **0.919** | -0.281 | **0.908** | **0.652** | **0.875** | **-0.519** |
|  | ***L_max_*** | 0.85 | 0.98 | 0.80 | 0.07 | 0.00 | NA | -0.212 | **0.844** | **0.524** | **0.844** | **-0.465** |
|  | ***K*** | 0.89 | 0.01 | 0.30 | 0.06 | 0.15 | 0.28 | NA | **-0.410** | **-0.442** | -0.274 | 0.248 |
|  | ***L_inf_*** | 0.33 | 0.32 | 0.94 | 0.05 | 0.00 | 0.00 | 0.03 | NA | **0.631** | **0.877** | **-0.478** |
|  | ***A_m_*** | 0.30 | 0.33 | 0.75 | 0.00 | 0.00 | 0.00 | 0.02 | 0.00 | NA | **0.726** | **-0.495** |
|  | ***L_m_*** | 0.06 | 0.66 | 0.26 | 0.10 | 0.00 | 0.00 | 0.16 | 0.00 | 0.00 | NA | -0.323 |
|  | ***M*** | 0.67 | 0.95 | 0.98 | 0.00 | 0.01 | 0.02 | 0.22 | 0.01 | 0.01 | 0.11 | NA |

**Table S2.2** Eigenvalues and percentage variation explained by the seven principal components axes using life history traits and spawning behavior parameters for the 28 species, and eigenvectors for each of the seven measurement indices.

|  | Principal component | | | | | | | | | |
| --- | --- | --- | --- | --- | --- | --- | --- | --- | --- | --- |
| Measure | 1 | 2 | 3 | 4 | 5 | 6 | 7 | 8 | 9 | 10 |
| Eigenvalues | 2.083 | 1.498 | 1.168 | 0.890 | 0.738 | 0.557 | 0.478 | 0.340 | 0.238 | 0.192 |
| Variation explained (%) | 0.434 | 0.221 | 0.136 | 0.080 | 0.055 | 0.031 | 0.023 | 0.010 | 0.006 | 0.004 |
| Cumulative variance (%) | 0.434 | 0.655 | 0.791 | 0.871 | 0.926 | 0.957 | 0.980 | 0.990 | 0.996 | 1.000 |
| Eigenvectors |  |  |  |  |  |  |  |  |  |  |
| Agg | -0.141 | 0.495 | -0.382 | 0.290 | -0.280 | 0.208 | -0.216 | 0.572 | -0.004 | 0.098 |
| Duration | -0.082 | -0.473 | -0.148 | 0.582 | -0.554 | 0.044 | 0.067 | -0.281 | 0.100 | 0.087 |
| Rel. Ab. | -0.013 | 0.567 | -0.402 | 0.019 | 0.022 | 0.003 | 0.274 | -0.664 | 0.006 | 0.025 |
| *A_max_* | -0.215 | -0.327 | -0.443 | -0.471 | -0.025 | 0.614 | -0.021 | -0.035 | 0.063 | -0.214 |
| *W_max_* | -0.407 | 0.131 | 0.267 | -0.025 | -0.002 | 0.066 | -0.747 | -0.345 | 0.232 | 0.100 |
| *L_max_* | -0.417 | 0.067 | 0.318 | -0.083 | 0.002 | 0.240 | 0.498 | 0.101 | 0.301 | 0.555 |
| *K* | 0.271 | 0.200 | 0.329 | -0.425 | -0.737 | 0.101 | 0.002 | -0.070 | -0.199 | 0.006 |
| *L_inf_* | -0.442 | 0.029 | 0.218 | 0.195 | 0.077 | 0.197 | 0.095 | -0.063 | -0.797 | -0.159 |
| *A_m_* | -0.345 | -0.169 | -0.369 | -0.356 | -0.149 | -0.602 | -0.067 | 0.051 | -0.251 | 0.367 |
| *L_m_* | -0.446 | 0.113 | 0.102 | -0.047 | -0.207 | -0.323 | 0.233 | 0.107 | 0.323 | -0.677 |


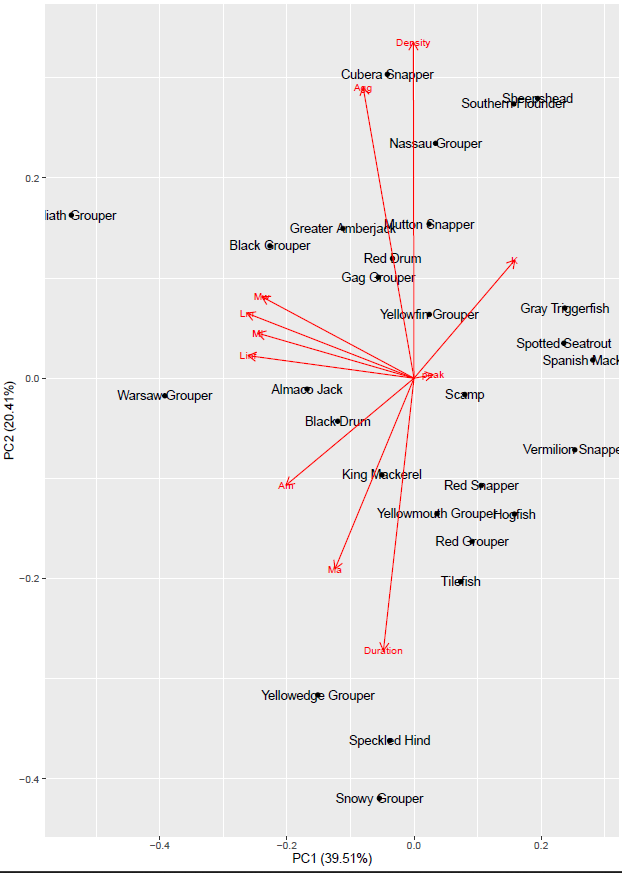


**Figure S2.1** Biplot of PCA showing the first two principal components and including Peak Spawning Months. The arrows show the relative loadings of each PC axis.
